# Ethical, Legal and Social Issues in Japan on the Determination of Blood Relationship via DNA Testing

**DOI:** 10.1007/s41649-017-0009-9

**Published:** 2017-06-30

**Authors:** Waki Toya

**Affiliations:** Research Fellow in the Office for Research Ethics and Bioethics, Research and Development Initiative Center, National Cerebral and Cardiovascular Center (Japan), Fujishiro-dai 5-7-1, Suita, Osaka, 565-8565 Japan

**Keywords:** Artificial Reproductive Technology, DNA testing, Ethical, Legal and Social Implications (ELSI) of Genetic Testing, Direct-to-Consumer Testing, Parent-child relationship

## Abstract

DNA paternity testing has recently become more widely available in Japan. The aim of this paper is to examine the issues surrounding (1) the implementing agency, whether the testing is conducted in a commercial direct-to-consumer (DTC) setting or a judicial non-DTC setting, and (2) the implementation conditions and more specifically the legal capacity of the proband (test subject). Literature research in Japanese and English was conducted. Some countries prohibit commercial DNA testing without the consent of the proband or her or his legally authorized representative. But as in some cases, the results of DTC paternity testing have proven to be unreliable. I propose a complete prohibition of DTC DNA paternity testing in Japan. In many cases of paternity testing, the proband is a minor. This has led to debate about whether proxy consent is sufficient for paternity testing or whether additional safeguards (such as a court order) are required. In cases where commercial DNA testing has been conducted and the test results are produced in court as evidence, the court must judge whether or not to admit these results as evidence. Another important issue is whether or not paternity testing should be legally mandated in certain cases. If we come to the conclusion that DNA test results are the only way to conclusively establish a parent-child relationship, then our society may prioritize even more genetic relatedness over other conceptions of a parent-child relationship. This prioritization could adversely affect families created through assisted reproductive technology (ART), especially in situations where children are not aware of their biological parentage. This paper argues for a complete prohibition of DTC DNA paternity testing in Japan, and highlights that broader ethical and legal deliberation on such genetic services is required

## Introduction

With DNA paternity testing, a scientific expert has the role of confirming blood relationship between a parent and a child. Its mounting popularity in recent years has resulted in the questioning and evaluation of the accuracy of DNA analysis, including the genetic polymorphism test that indicates the presence or absence of a blood relationship.

Many societies have tried to regulate genetic testing, and many governments have issued guidelines. However, in Japan, DTC (direct-to-consumer) paternity testing has not been discussed much yet. So far, DTC testing has mainly been discussed with regards to hereditary disease and socially desired traits, such as enhanced athletic capacity, although testing in the latter sense is not scientifically reliable (Samuel et al. 2010, Wagner and Royal 2012).

In this paper, I first provide an overview of DNA paternity testing in Japan. Second, I highlight the problems caused by a lack of appropriate governance of commercial companies offering DTC testing, especially when test samples are taken without proper consent or are taken in secret. Third, I problematize proxy consent (both in judicial and extrajudicial testing) and the voluntariness of DNA paternity testing. In this context I clarify when the courts or the law could mandate paternity testing. Fourth and finally, I discuss the influence of a growing popularity of DNA paternity testing for the parent-child relationship in the context of certain forms of Assisted Reproductive Technology (ART).

Topics not covered in this paper include prenatal paternity testing or disclosure of incidental non-parentage discovery in clinical settings, such as during preparation for a living donor transplantation or during DNA testing for genetic disease, as described by Young (2009).

## An Overview of Parentage Determination via DNA Testing in Japan

Omura (2003) separates issues surrounding DNA paternity testing in Japan into two major categories: issues concerning (a) the implementing agency and (b) the implementation conditions (consent-related).

### A classification of implementing agencies

DNA testing agencies in Japan include both public (judicial) and commercial entities. Such commercial DNA testing entities (which may also test for disease or for disease susceptibility) are grouped into three different types by a report commissioned by the Ministry of Economy, Trade and Industry (METI 2013): (1) DTC companies that send a test kit to consumers, who collect DNA samples themselves, (2) non-DTC companies that use medical professionals or scientific experts to collect the DNA samples and (3) companies using both DTC and non-DTC models. Most of these commercial entities are DTC companies typically only accepting online orders.

Both DTC and mixed DTC/non-DTC companies, as classified by METI, usually offer disclaimers on their website that DTC testing cannot be used as evidence in lawsuits. It is not yet clear whether the courts treat DTC testing as conclusive evidence of genetic and familial relationship or not, although there is an obvious potential for customers of DTC companies to subsequently initiate legal proceedings based on their test results.

### Implementation Conditions and the Challenge of Voluntary Consent

The conditions under which genetic testing are implemented are mainly concerned with the legal capacity of the proband and the agreement of other people that are involved in the procedure. The legal and ethical issues that underlie these conditions include propriety of samples taken from minors (as well as issues that relate to proxy consent), samples taken without the permission of the proband or legally authorized person (secret testing) and, in the event of civil trials, the voluntariness of the persons submitting samples for testing.

**Fig. 1:**
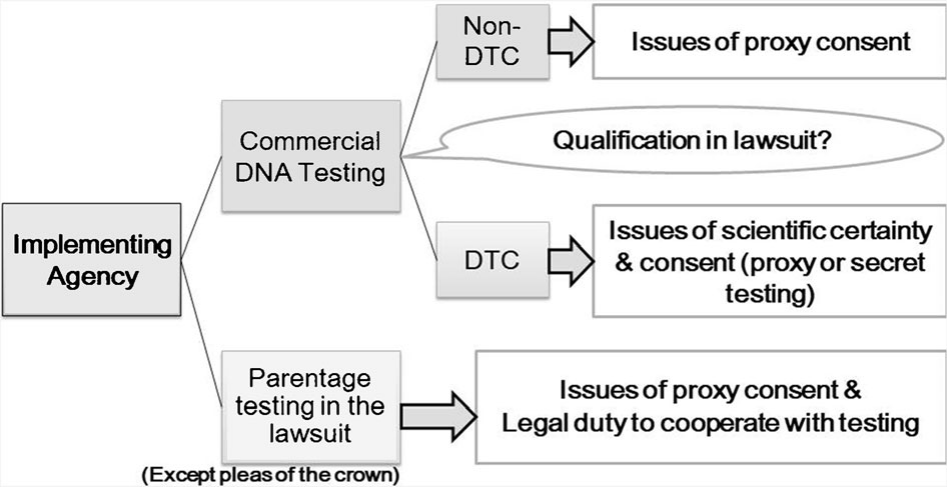
Voluntary consent as a key concern

Figure 1 shows the ethical and legal issues surrounding consent and voluntariness that arise depending on the type of testing agency. Specifically, concerns over the voluntariness and appropriateness of proxy consent apply to both commercial DNA testing and parentage determination in legal proceedings. In brief, there are more serious concerns over voluntariness and ethical appropriateness of commercial DTC testing because of issues of scientific reliability, inadequacy of proxy consent in protecting the welfare of minor probands and issues of secret testing. In the next section, I will discuss ethical concerns about scientific reliability and secret testing in commercial DTC DNA testing.

## Issues of Commercial DNA Testing Agency in Japan

### Commercial Paternity Testing

In recent years, genetic testing has expanded rapidly in Japan. Since 2000, the Japan Registered Clinical Laboratories Association (an industrial association on clinical examination in Japan) has surveyed the number of DNA and chromosome tests conducted by its approximately 120 members, via a bi-yearly questionnaire. The numbers of almost all types of DNA testing have increased each time the survey was conducted (Japan Registered Clinical Laboratories Association 2016). Although the number of members of this association is relatively limited, no similar surveys have been conducted in Japan. According to the METI report (2013, pp. 17–22), only a few companies test samples themselves domestically, most companies however—often smaller clinics and pharmacies—send their samples abroad for DNA testing. The METI report does not specify the proportion of testing that was done for establishing parentage or for other health-related purposes. However, the report mentions that most common DTC genetic testing conducted by foreign DNA testing companies, was for parentage determination.

On websites of Japanese companies offering both DTC and non-DTC parentage determination, non-DTC testing fees are at least twice as high as DTC fees. Some companies sell DTC tests as a ‘simple examination’, costing from 10,000 to 20,000 yen (or US$88–175, US$1 = JP¥113.7 on 12 May 2017) and advertising it as a ‘low cost, high accuracy and easy procedure’ (Gene track Japan 2017). One company provided over 150 paternity test results per month. The head of this company said that requests have increased because of decreasing cost and increasing public knowledge of such a service, after a scandal involving a celebrity family, and DTC paternity testing got publicized (AERA 2016). At this company, 40% of customers were reportedly ‘fathers’, whereas 40% were ‘mothers’ and 20% were ‘grandparents’. Some couples who had children through in vitro fertilization also wanted to confirm the parentage of their children through such tests. In Japan, the proportion of children born via assisted reproductive technology (ART) has increased throughout the 2000s to about one in 21 babies in recent years.

For comparison, the ‘recreational’ genetic ancestry testing is not common in Japan. So the possibility of accidental findings of non-paternity by the result of DTC ancestry testing (Moray et al. 2017) have never been realistic matter.

Commonly, non-DTC samples are taken at dental clinics on behalf of commercial DNA testing facilities in Japan. Although almost all clinics reported that they do take the consent from the proband or a legally authorized person about the testing, they are not involved in explaining the procedure in detail (METI 2013, 36). Dental clinics use the consent forms made by associated commercial DNA testing facilities and just tell the probands to sign it. These clinics think that it is the responsibility of the testing facility to properly inform and counsel the proband and/or legally authorized persons concerned. They just collect the sample and earn commission for the service that they provide.

### Misinformation in Commercial DTC Parentage Determination

In commercial paternity testing, both DTC and non-DTC facilities must confirm the identity of the proband and ensure that appropriate consent or permission has been obtained. In non-DTC testing, there is lower risk of secret testing, as samples are collected by staff of (dental) clinics, and DNA testing facilities take consent from the proband or his or her parent or guardian. DTC testing, however, involves greater risks concerning lack of voluntariness and misinformation (Mertens 2006).

Some of the websites of DTC testing companies advertise the ability to determine parentage from hair, hair follicles, nail cuttings, or tissue paper containing nasal discharge (Non-profit Organization Gene Information Analysis Center 2017). They call these ‘special samples’ that could be utilized if the consumer cannot obtain a sample of a person’s mucous membrane from the oral cavity, as is customary in testing. There is no mention of consent or permission of the proband from whom such samples have been obtained. Some researchers in Japan have criticized this practice since the 1990s (Sato 2012). Obviously, such ‘secretly picked up’ samples cannot be objectively identified, and the test results of such secret sampling readily becomes a source of legal and psychological conflict within families.

Apart from concerns over scientific reliability of the test results, lawsuits against commercial DTC paternity testing facilities detail wrongful practices occurring when testing the sample, analysing the data and translating the results from foreign languages to Japanese (Tanamura 2014). The METI report cites a case involving DTC testing in which ‘different results were shown from the same sample tested at two different agencies. Quality of inspection issues were revealed.’ (METI 2013, pp. 153–154)

Although DTC companies clearly states that DTC test results cannot be used as evidence in court, the handling of these data (even as a reference) in lawsuits is a legal problem because Japanese law makes no mention of the handling of paternity test results obtained without the permission of the party concerned (Sato 2012, p. 51).

### The Sequence of Possible Outcomes Resulting from Secret Paternity Testing

**Fig. 2:**
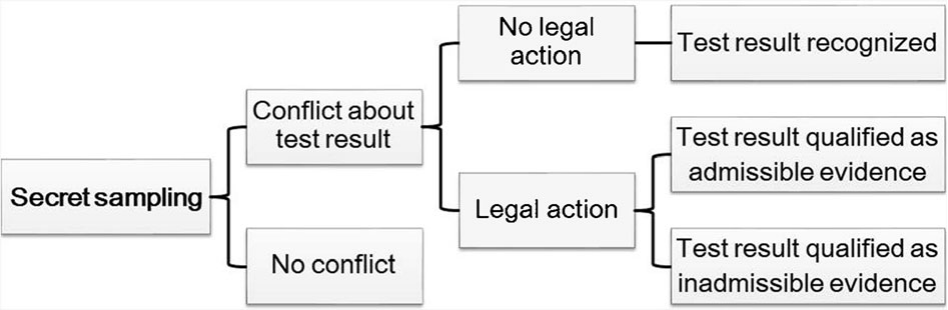
Possible outcomes from secret sampling and testing

Figure 2 shows the sequence of possible outcomes after a consumer secretly collects a sample for paternity testing by a commercial facility without the permission of the proband or his/her legal guardian.

According to Fig. 2, four outcomes are possible. First, ‘no conflict’ may ensue if the interested parties find the test result acceptable and decide not to take any further action. This may be the case, either when the result does not change existing familial arrangements and accepted blood relationships, or when the result is subsequently concealed in order to avoid contestation. If conflict arises from the test result, ‘No legal action’ is used to refer to a second outcome where the conflict is settled out of court by the interested parties, and the parties recognize the result of DTC testing as accurate. If legal action is taken, the court would have to decide whether to qualify the DTC paternity test result as admissible or inadmissible evidence (third and fourth outcome). However, courts have generally held a negative view of legal utilization of paternity test results of samples taken secretly without the consent of the appropriate parties.

A working group on DNA parentage testing in the Japanese Society of Legal Medicine developed a guideline for parentage determination (1999), which stipulates the following: ‘A genetic testing facility should ensure there is no objection to the testing among the parties or donors of the samples’, and ‘Expert witnesses must not test a sample whose collection status is unconfirmed’. Moreover, the group mandated the recording and archiving of the signature of the proband or a legally authorized representative.

Some countries, especially in Europe, currently prohibit secret DTC testing and have criminal penalties in place to enforce the prohibition (Toyoda 2007). Justifications for the prohibition against secret DTC paternity testing include concerns about the reliability of samples taken without the permission of the proband, the ethical issue of genetic testing without consent and the negative impact of test results on innocent parties involved. Therefore, in countries like the United Kingdom, DNA testing without appropriate consent is an offence that ‘could lead to a fine, a term of imprisonment of up to 3 years or both’ (Human Tissue Authority 2017). In addition, a paternity test result that is obtained illegally could be inadmissible as evidence in legal proceedings.

### Restriction on Commercial Parentage Determination

As mentioned above, it is unclear whether robust regulations and mechanisms are in place to ensure that appropriate consent has been obtained for DTC paternity testing by a commercial facility, and even in a non-DTC setting. Because genetic testing is broadly recognised to draw many ethical issues and the results may be difficult to interpret correctly, many countries emphasize the importance of genetic counselling before and after testing. It is also difficult to regulate when DTC paternity testing is conducted as an international commercial service.

Because there are so many hazards involved in commercial DTC paternity testing, I argue that its provision as a commercial DTC service should be prohibited. Mertens (2006, p. 75) asserts that the core ethical issue of ‘parentage testing is the reality of secretly or abusively performed paternity tests’. DTC parentage testing is more problematic than other DTC testing applications, such a DNA testing which advises about effective fitness or diet to lose weight, because commercial DTC paternity testing could have an adverse impact on an innocent proband, who would typically be a minor.

Commercial DTC paternity testing without counselling jeopardizes families with psychological and legal issues. Genetic counsellors in Japan tend to think they should only counsel about genetic disease and medical treatment, and that DNA parentage testing is not part of their work because DNA parentage testing is not a diagnosis of disease. On the other hand, some forensic doctors (who have historically been ordered by the courts to conduct DNA testing even in civil disputes) involved in court-ordered parentage testing need lawyers or ‘consultants’ to conduct legal or genetic counselling in order to resolve domestic disputes which would typically arise when parties to the dispute are required to undergo DNA testing. Where children are involved, domestic conflicts may worsen as children exhibit signs of tension and stress (Araki 2014, p. 104; Katsumata 2014, p. 210–222). As a consequence, even if no new information emerges from DNA testing, the demand for testing itself could have a deep psychological impact on the parties involved. For minors or children in particular, seeing a conflict between relatives over their status would be damaging to their wellbeing. Thus, we must seriously consider the limitations of proxy consent in paternity testing.

## The Issue of Proxy Consent in Judicial Testing

### Restrictions on Proxy Consent About DNA Testing

Is it reasonable to carry out DNA testing without consideration of a person’s ability to consent? In paternity testing, the proband is usually a minor. This has led to discussion about the appropriateness of proxy consent in paternity testing, and whether it should be supported by a court order or not.

The METI report reviewed domestic and foreign guidelines on DNA testing (METI 2013, pp. 107–130). Many foreign guidelines for genetic testing, including DNA paternity testing, recommend confirming the capacity for consent (except in special circumstances) of the proband or proxy and, ideally, the postponement of testing for minors until they reach the age of majority. The Austrian Bioethics Commission has strongly recommended that parents or legal guardians must refrain from DTC (online) genetic testing with samples collected from minors or incompetent persons. In Belgium, a proposal was laid down for a law to regulate paternity testing, mainly by limiting testing to a child under 1 year old, or the first 4 years of his or her adult life (Toyoda 2007, p. 95; Mertens 2006, p. 76). Mertens (2006) mentioned the proposal had included consent from both parents, additional limitations on facilities that can conduct DNA paternity testing and psychological assistance, etc. However, this proposal has not been implemented in Belgium (Borry et al. 2012). Elsewhere, other restrictive (legal) procedures on DNA paternity testing have also been proposed if the proband is a minor (Caenazzo et al. 2008; Bird 2014).

Two guidelines in Japan discuss the minor’s welfare in cases of paternity testing. The Japanese Society of Legal Medicine (1999), (1) only recommended that: ‘You should pay maximum attention to the welfare of children, who tend to have little voice’. Another guideline by METI ( 2004, p. (13) on general DNA testing notes:

a point to consider regarding DNA testing and parentage testing … You must take care about these points: (1) You should pay maximum attention to minors’ welfare, especially that of infants and toddlers, who tend to have little voice; (2) You must be mindful that there are no objections to the testing among parents, children, or other donors of samples, who are directly affected by the test.

While some commercial DNA testing agencies follow the METI guidelines, there is no way to confirm how the interests and welfare of minors in Japan who undergo paternity testing are safeguarded.

### The Supreme Court Decision on Commercial DNA Paternity Test Results in Japan

Toyoda (2007, pp. 81–82) noted that some lawsuits in Japan have attempted to treat the results of commercial non-DTC paternity testing as evidence. In 2014, the Supreme Court decided three cases involving private DNA testing on the same day. They represent the first decision by the Supreme Court on the case involved commercial DNA paternity test results. While the Supreme Court ruled against the use of these commercially obtained paternity test results, it did not make any pronouncement on the legal admissibility of the results as evidence in all three cases.

In one commercial paternity testing case (Sup. Ct, 2014a, No. (O)226), the plaintiff was a husband who subjected his two children (of uncertain age at the time of testing) to paternity testing. The test result was rejected as untrustworthy by the defendant who was his wife. The defendant won the case because the lawsuit was dismissed due to the expiration of the statute of limitations. The other two cases (Sup. Ct, 2014b, No. (Ju)233 and Sup. Ct, 2014c, No. (Ju)1402 were similar to each other and had similar judgements. The plaintiffs were wives who conduct paternity DNA testing on their children, who were about 2 years old in both cases at the time of testing. Both women wanted to be divorced from their defendant husbands, but were unsuccessful. The Supreme Court prioritized the legal status of the child in ruling that a legitimate parent-child relationship existed. The rationale was based on Japan’s civil law, in which ‘the husband of the woman who bore the baby is presumed to be the father of the baby’. (Minpo [Civil Code], art. 772, para. 1)

It is unclear whether the tests in these cases were DTC or non-DTC; however, the reliability of the test in the latter two cases was not contested. In these three cases, the courts did not require the paternity testing to be repeated. In the first of these cases (i.e. No. (O)226), the defendant wife contested the test result as unreliable. The Supreme Court decision did not comment on the reliability of paternity testing but made its judgment based on technical legal grounds. In contrast, one of the judges in a supporting opinion in the decision of case No. (Ju)233 expressed the need for a law on how to treat and admit paternity test results as evidence and cautioned that the test results must not take precedence over the welfare of the child. The decisions in all three cases reflected the Supreme Court’s concern over the potential abuse that commercial paternity testing presents, particularly in its reluctance to lend any weight to the results adduced by the plaintiffs. At the same time, none of the judgments in these cases mentioned proxy permission or the issue of minors lacking the capacity to consent to DNA testing.

### Welfare of the Child and Proxy Consent

Parents or legal guardians are stakeholders in this issue, as they have the responsibility of providing proxy consent for testing based on what is in the best interests of the child concerned. Regardless of whether or not paternity test results are presented in legal proceedings, questions remain about proxy consent for paternity testing: Is the use of samples from a minor or a person without decision-making capacity? Does proxy consent by parents protect the child’s moral rights and welfare? And how should we protect the child’s moral rights and welfare? All of these questions are important ethical and legal considerations in proxy decision-making.

I make the point that the testing of samples from a minor or incompetent person must be restricted, as it is in other western countries, because the result could adversely impact the proband’s wellbeing and mental development. Some children may grow up regretting that they had been too young to refuse the test. Thus, the restriction on paternity testing in Japan should be restricted by the age of the child involved. Based on Japanese case law, if a married couple has no possibility of having a child (for example, due to separation, imprisonment or infertility), the presumption that ‘the husband of the woman who bore the baby is … the father of the baby’ (Minpo [Civil Code], art. 772, para. 1) is not applicable. In addition, the father could file a lawsuit to deny the legitimacy of his child within a year after he knows of the child’s birth (Minpo [Civil Code], from art. 774 to art. 778). The time limit of 1 year seems reasonable for the child and the father to establish a stable relationship. It seems that under Japanese law, the presumption of parentage would apply if a parent does not legally challenge the parent-child relationship on the basis of lack of genetic relatedness. In a subsequent case of switched babies in Japan, a nonfiction writer reported that: ‘Half of parent-child relationship is DNA (genetic) relationship, the other half is attachment between them’ (The Asahi Shimbun (Newspaper) 2014). Genetic relatedness matters, but not absolutely.

In Section 3.2, Case No. (O)226 illustrates how paternity test result was resisted by the defendant in that case. I question whether proxy consent of only one parent should be sufficient for paternity testing. The 2004 METI guideline states that there must be no objection among interested parties, but the guideline is not legally enforceable. In addition, another problem is with the one-sided nature of proxy consent. Moray (Moray et al. 2017) highlights this challenge in the context of ancestry testing, which is becoming a growing market. And even if one parent consents to parentage testing, the test result may disclose genetic information of the non-consenting parent (Barrot et al. 2014).

Proxy consent by a parent could also run counter to the child’s moral rights and welfare. The Japanese Civil Code (Minpo [Civil Code], art. 826, para. 1) has an article on resolving conflicts of interest (COI) between a parent and a minor. If a parent wants to act in a manner that presents a COI with the child, the parent must request for a family court to appoint a procurator for the child. If the parent’s motivation for paternity testing runs counter to the child’s welfare and interest, this article should apply.

An opinion by a judge in Case No. (Ju)233 stated that children should have the right to know their parentage once grown and had attained sufficient maturity and understanding and should have the choice to exercise that right. I support this opinion. As an adolescent, a minor can understand the significance of paternity testing and could voice his or her own opinion on whether to undergo the test or not. This is consistent with the recognition that an adolescent should have the right to express his or her thoughts about research participation and medical care. Many studies on informed consent recommend involving minors over 14 years of age in decision-making, with some assistance (Kuther 2003; Weithorn and Campbell 1982). In Japan, there is no law that specifically indicates when minors have the capacity to make medical decisions independently of their parents or legal guardian. Studies about informed assent from children suggest that age should not be definitive of this. The age of majority in Japan is 20 years, although marriageable ages are 16 years for a female and 18 years for a male. A married minor is treated as an adult in civil law. Government ministries and civil societies differ in their guidelines when a minor could consent to medical treatment. Because a minor is indicated as having testamentary capacity from 15 years of age in the Japanese Civil Code, many lawyers and the Health, Labour, and Welfare Ministry (1997) recommend that a minor of that age could also consent to medical treatment. Most lawyers also think that a person aged 15 years and above should have a right of self-determination because it is the end of compulsory education and also the eligible age for a person to be granted the right to work under the Labour Standards Act. Where research is concerned, a person who is 16 years and above has the capacity to provide informed consent under the ‘Ethical Guidelines for Human Genome/Gene Analysis Research’ (2014) and the ‘Ethical Guidelines for Medical and Health Research Involving Human Subjects’ (Ministry of Education, Culture, Sports, Science and Technology, Ministry of Health, Labour and Welfare, and Ministry of Economy, Trade and Industry 2014).

Some researchers recommend that a minor who has been under long-term stress from an illness should have the maturity and understanding to be involved in the provision of informed consent to participation in research concerning the illness. I do not consider paternity testing to be a form of medical treatment or research, but its impact could resemble that of long-term stress or illness. Consequently, the decision of a mature minor with sufficient understanding of the implications of paternity testing should be recognised as having the legal capacity to decide on whether to undergo the test or not.

These Supreme Court judgments reflect a position that respects privacy and supports the right to self-determination. The opposing opinion represents the position that confirmation of blood relationship is the biggest benefit and welfare for a child. Such an opinion links the thought that refusing to undergo paternity testing could be viewed as an attempt to conceal one’s genetic identity or status. We consider this opinion further in the sections below.

## The Issue of the Legal Mandatory Power of DNA Parentage Testing

In Japanese family law and case law, the refusal or non-cooperation of a party to undergo paternity testing cannot lead to a prejudicial outcome for the opposite party, and according to one popular legal theory, the court cannot force a party to undergo paternity testing (Sato 2012, p. 51). In fact, there are examples where a request by the court for DNA testing was refused (the Tokyo High Court, 30 Jan. 1995). Thus, introducing a legal obligation to undergo paternity testing has been discussed since the 1950s in Japan (Toyoda 2007, p. 87).

Elsewhere, there is an increasing number of legislations that mandate paternity testing under certain conditions. For example, in family law in South Korea, a person must pay penalty charges if she/he refused parentage testing but has been ordered to do so by the court (Kim 2012). Also, the USA has taken an active interest in DNA testing to determine the fathers of children born outside marriage because of the need to cut the welfare budget (Yano 1996).

Should we legislate such an obligation? Opponents say that the system to evaluate a child’s legitimacy is meaningful because ‘a father bears the rearing responsibility for the child and thus should have the right to establish the child’s genetic status’. In addition, the ease of paternity testing has rocked the *raison d’être* of family law, which seeks to protect the welfare of the child, since the enforcement of paternity testing could cause more harm than good (Miyake 2003). Moreover, advocates have suggested that the introduction of paternity testing would eliminate the courts’ need for proof of indirect facts involving personal matters (such as unfaithfulness); they also argue for a child’s right to know his or her parentage, which has also been discussed in relation to assisted reproductive technology (ART) and gamete donation.

## The Influences of Expanding Paternity Testing in Assisted Reproductive Technology

According to Japanese case law, the mother is the woman who delivered the baby (Sup. Ct., 27 Apr. 1962). Most cases of parental determination relates to establishing paternity, although there was one case where maternal status was denied because of DNA testing. Although the child has lived with the mother for many years, she was secretly adopted and registered as if she had a blood relationship with the mother (The Tokyo High Ct. 26 July 2005). In case No. (Ju)1402, the Supreme Court rejected the result of the DNA parentage testing. One of the reasons for the ruling was that the child’s father had acknowledged that the child was not genetically related to him from the beginning but he has accepted the child as legally and socially his own from her birth. In this way, the courts in Japan have distinguished between social (or actual lived) relationship from a genetic one.

Sasaki (2007) considers the issue of gamete donation for ART as a ‘new type of secret adoption’. To summarize, both secret adoption and gamete (embryo) donation for ART share common issues: ‘the legal fiction of blood relations’, ‘the actual social conditions as parent and child,’ and ‘the instability of the parent-child relationship brought about by the introduction of DNA testing’ (Ravelingien and Pennings 2013; Turney 2010).

The expansion of commercial DTC DNA testing could influence the rights of children born through gamete donation to know their parentage. Most Japanese parents who undergo ART with third party gametes are reluctant to tell their children about their genetic origins. However, it is possible for children to learn this through commercial DTC paternity testing. Thus, the parents are not the only way to know the truth. If Japanese legislation gave free rein to online commercial DTC paternity testing kits, a child of sufficient maturity and understanding could buy a DTC parentage testing kit online (even if done so just out of curiosity) and get a shocking surprise without any psychological support. She/he should have the right to know her or his blood relationship though, provided that proper psychological support is in place. Otherwise, easy access to DTC parentage testing kit could result in greater harm to the child.

## Conclusion

In this paper, I have presented the problems surrounding DNA paternity testing and their effects on society and argued that commercial DTC paternity testing should be prohibited. To protect the welfare of children, such testing must not be regarded as a simple business opportunity that contributes to economic development.

The essential characteristics of a parent-child relationship include the ‘intention to form a parent-child relationship, the existence of the actual lived experiences as parent and child, the representations of a parent-child relationship (in law and society)’ (Miyake 2003, 56) as well as genetic relations. Which of these criteria determine the parent-child relationship? The expansion of DNA paternity testing threatens to prioritize genetic relatedness over other expressions of parent-child relationship. Although issues related to DNA testing have received some legal attention in Japan, broader ethical and legal deliberation is required with the increasing availability of DTC paternity testing services.
